# Physical activity of community-dwelling adults with traumatic spinal cord injuries in the Cape Metropole

**DOI:** 10.4102/sajp.v81i1.2147

**Published:** 2025-06-13

**Authors:** Aeysha Gabriels, Toughieda Ismail, Lucian Bezuidenhout, Conran Joseph

**Affiliations:** 1 Department of Physiotherapy, Faculty of Community and Health Sciences, University of the Western Cape, Cape Town, South Africa; 2 Division of Physiotherapy, Department of Health and Rehabilitation Sciences, Stellenbosch University, Cape Town, South Africa; 3 Division of Physiotherapy, Department of Neurobiology, Care Sciences and Society, Karolinska Institutet, Stockholm, Sweden

**Keywords:** traumatic spinal cord injuries, community-dwelling, physical activity, sedentary behaviour, LIPA, MVPA, Western Cape

## Abstract

**Background:**

Physical activity (PA) is an important indicator for disease prevention in adults with spinal cord injuries (SCIs). Regular PA can improve functioning, community reintegration, economic participation and overall well-being.

**Objectives:**

Determine PA levels of community-dwelling adults with traumatic spinal cord injury (TSCI) in the Cape Metropolitan, South Africa.

**Method:**

A quantitative, cross-sectional design was used in the Cape Metropole. The population included all community-dwelling adults with TSCIs, regardless of mobility status. Physical activity levels were measured using an ActiGraph wGT3X-BT accelerometer, classified by intensities: sedentary behaviour (SED), light intensity PA (LIPA) and moderate-to-vigorous PA (MVPA). Descriptive and analytical statistical tests described PA intensities and investigated group differences.

**Results:**

A total of 76 participants (mean age 36 years, standard deviation [s.d.] 10.93), mainly males (88.2%), were recruited. Time spent in SED, LIPA and MVPA among wheelchair users was 761.12 min/day (77.6%), 203.11 min/day (20.7%) and 16.96 min/day (1.7%), respectively. Ambulatory individuals spent 972.47 min/day (98.1%) in SED/LIPA and 18.80 min/day in MVPA (1.9%). Time since injury (*p* = 0.005) and age (*p* < 0.001) resulted in more MVPA for older wheelchair users and ambulatory individuals with recent injuries.

**Conclusion:**

Participants spend most time sedentary, followed by LIPA. Adults with SCI are not meeting recommended PA levels for health benefits. Understanding barriers to PA is essential for developing targeted interventions to optimise PA levels.

**Clinical implications:**

Cape Metropole’s unique SCI profile with mostly young males, results in long-term injury impacts. Sedentary behaviour increases risks for morbidity and early mortality. Thus, exploring PA’s role in SCI rehabilitation is important for healthy ageing.

## Introduction

In developing countries, spinal cord injuries (SCIs) are currently a public health concern (Jesuyajolu et al. 2023). In 2021, the World Health Organization (WHO) estimated that over 15 million people currently live with an SCI worldwide (WHO 2024). In South Africa, the incidence rate for traumatic spinal cord injury (TSCI) was estimated at 76 per million people, which was found to be one of the highest when compared to developed, Western countries (Joseph et al. 2015). Globally, adults with SCIs often lead very sedentary lifestyles, and this predisposes them to develop secondary complications and puts them at an increased risk for developing cardiovascular disease (CVD), diabetes and obesity (Cragg et al. 2013; Garcίa-Massó, Serra-Añó & Gonzalez 2015; Nooijen et al. 2015; Warms, Whitney & Belza 2008). In the previous literature, it was noticed that there is a 17% prevalence of CVD among the SCI population compared to a 5% prevalence among the non-SCI population (Cragg et al. 2013). Physical activity (PA), however, is an important modifiable factor in decreasing the risks of CVD, stroke and diabetes (Cragg et al. 2013; Garcίa-Massó et al. 2015; Nooijen et al. 2015; Warms et al. 2008).

The PA, defined as bodily movement that results in energy expenditure, can be classified in frequencies, intensities and step counts (Ainsworth et al. 2000; Park et al. 2021; Veerubhotla et al. 2020). It is well known that the lack of activity devastates health status. Physiological detriments such as pain, fatigue, slower metabolism, muscle deconditioning and atrophy are often associated with prolonged bed rest (Warms et al. 2008). They may further hinder the ability to engage and optimise one’s PA. Engaging in PA early after the injury may be a powerful and important target for rehabilitation programmes to prevent primary and secondary consequences (Soriano et al. 2022). In the Western Cape Province of South Africa, there is only one inpatient facility, offering specialised services for adults with SCIs where not all patients are admitted for care. The remaining patients are further managed at primary and/or community healthcare centres where there are currently no guidelines for the prescription of rehabilitation (Joseph et al. 2015). Furthermore, physiotherapy rehabilitation services are not available at all primary healthcare facilities, with recent inquiries indicating the availability of either physiotherapy or occupational therapy at less than 10% of the primary healthcare level in the specific setting. The continuity of care across all levels of care is suboptimal because of poor and inadequate referral processes (Conradie, Berner & Louw 2022; Louw et al. 2023). This highlights the lack of sufficient resources available for rehabilitative services for adults with SCIs and that potential innovative approaches to the engagement of PA may not be a top priority for these healthcare workers.

The WHO has recommended PA guidelines for abled-bodied individuals, as well as adults with SCIs to decrease morbidity and mortality rates (Martin Ginis et al. 2018). These guidelines are for cardiometabolic and health fitness benefits and entail approximately 30 min of moderate to vigorous exercise three times a week (Martin Ginis et al. 2018). However, in developed countries, a recent systematic review reported that 27% – 64% of participants do not engage in any PA (Stendell et al. 2024), and there is currently no evidence available in South Africa that quantifies PA in adults with TSCIs. As PA is complex, an individual’s ability to engage in PA is typically driven by the motivation to obtain health benefits by evoking behaviour change or the prescription of exercise based on need (Barron 2007; Greenman 1996; Rodrigues et al. 2023). The social cognitive theory of behaviour change has been commonly identified in the literature as driving the engagement of PA, which proposes that individuals are motivated to behave based on their intentions (Bandura 2001). However, there are several sociodemographic and injury-related factors, which may influence the ability of an individual with a TSCI to engage in PA. These include age and gender, where older males are more likely to engage in PA (Watson et al. 2022). Regarding injury characteristics, severity and time since injury also influenced PA, where complete lesions were more sedentary and participants who sustained their injuries for a longer time engaged in more PA. These factors must be considered when developing fit-for-purpose interventions to bolster PA engagement.

In developed countries, CVD has become a leading cause of death in adults with SCIs, which highlights the need to encourage a physically active lifestyle for adults with TSCI in South Africa. In addition, factors such as low-income living areas with high levels of crime and limited resources, from which a large proportion of adults with TSCI come and reside, are associated with restricted mobility, which is detrimental to long-term health and functioning for this population (Fyffe, Botticello & Myaskovsky 2011). Against the backdrop of these influencing factors, our study aimed to: (1) describe PA levels, in terms of time spent in different intensities, of community-dwelling adults with TSCI and (2) determine potential associations between demographic and injury characteristics and PA levels, that is, intensities. This information will aid in developing an understanding of how PA should be supported and augmented in the South African context.

## Research methods and design

### Setting

Our study was conducted in the Cape Metropole region of the Western Cape, South Africa. This district has two tertiary hospitals providing acute care for TSCIs, namely Groote Schuur Hospital (GSH) and Tygerberg Hospital. The GSH is the only facility with a specialised SCI unit providing acute care for adults with TSCIs in the Western Cape (Sothmann et al. 2015). As a result of limited resources (number of beds or placements), patients are admitted to these hospitals on a referral basis (Joseph et al. 2015). Specialised rehabilitation is offered at Western Cape Rehabilitation Centre (WCRC). Still, patients are seen here on a referral basis, and only patients who demonstrate the best propensity for recovery are accepted (Joseph et al. 2015). Limited information is available on the continuum of care post-discharge from the acute setting or whether patients are further managed at their local primary and/or community healthcare centres in the community.

### Design and participants

In this quantitative, cross-sectional design, participants were sourced from a GSH database for the years 2016–2020. The database consisted of all adults with TSCI who had been admitted during the selected period. The inclusion criteria included all participants (18 years and older) consenting to our study, those with TSCIs with lesions C6 and below (who will be able to mobilise with their wheelchair manually) and those adults with SCIs living in the communities of the Cape Metropolitan region for at least 1-year post-injury. The TSCI participants who presented with other neurological impairments or severe cognitive deficits that prevented them from providing consent or the ability to respond to relevant testing were excluded from our study. The STROBE reporting guidelines were used in planning and reporting the findings of this article.

Our study population was 297, based on the number of eligible adults in the database. The Yamane (1967) statistical formula was used to compute the sample size based on a 95% confidence interval, where 170 participants were required to yield statistical power. Ultimately, 76 adults with TSCI took part in our study, and a convenient sampling strategy was thus used as opposed to a probabilistic and representative sample. A large proportion (*n* = 214 of *n* = 297) could not be reached via their contact details; 10 relocated to other areas and provinces in South Africa, and 2 declined participation in our study. The final sample was deemed sufficient for an exploratory study of several risk factors linked to PA (Al-Habib et al. 2011). However, as seen with the pre-selected stratified analysis, the number of risk factors needed to be minimised to ensure sufficient analysis power and prevent Type I statistical errors.

### Data collection procedure

Data collection commenced in the year 2021, from November to August 2021. All eligible participants were initially contacted telephonically. Participants who could be reached telephonically were informed about our study, and arrangements were made to meet with them at a time and place that was convenient for them. Participants were either contacted telephonically or located at their homes in the community. The data collection process was twofold: participants were fitted with the accelerometer, and questionnaires were administered. This included a range of socio-demographic and injury characteristics, a secondary medical complications scale and the Spinal Cord Independence Measure – Self-report. The entire test battery took approximately 45 min to complete.

The PA levels were measured using an ActiGraph wGT3X-BT accelerometer. The ActiGraph accelerometer was initialised using the ActiLife software (version 6) before the assessment. The accelerometer was placed on either the wrist for wheelchair users (*n* = 56) or the ankle for ambulatory individuals (*n* = 13). Each participant was asked to complete a timesheet diary for 7 days, with the first day of data collection starting the day after the assessment. Participants were asked to log their wear time from when the device was fitted in the morning to the time the device was removed in the evening. Participants were informed always to wear the device but to remove the device when bathing or before any water activities (e.g. swimming) and when sleeping at night. The device was then collected on day 9, allowing for 7 days of data collection. Any participants who had invalid data, that is, < 3 days of 10 h of daily measurement were excluded from our study.

### Instrumentation and outcome measures

The socio-demographic and injury characteristics were prompted using the International Spinal Cord Injury Core Data Set version 2.0 (De Vivo et al. 2006). This instrument covers aspects related to injury date and cause, vertebral and other associated injuries, length of hospital stay and neurological classification (De Vivo et al. 2006). In addition, a self-made questionnaire was developed to obtain information on CVD risk factors, family history of CVD, current residence, employment history, educational history and marital status.

The secondary medical complications were assessed using the secondary health complications scale for SCIs (Kalpakjian et al. 2007). The assessment tool consists of 16 questions using a 4-point ordinal scale. This test has proven to be valid and reliable, with internal consistency exceeding 0.76 and test-retest reliability ranges of 0.56–0.80 (Kalpakjian et al. 2007). Furthermore, functional independence was assessed using the Spinal Cord Injury Measure – self-report (SCIM-SR) scale (Catz et al. 2001). This subjective self-report measurement assesses ADL function in self-care, respiration and sphincter management, mobility and transfers, with a maximum score of 100. The higher the scores, the more independent the individual is (Catz et al. 2001). This instrument is valid and reliable with an intraclass correlation coefficient of 0.83 for the entire scale (Bluvshtein et al. 2011).

### Objective measurement of physical activity

As stated above, the ActiGraph wGT3X-BT accelerometer was used to measure PA levels. The accelerometer is reliable and valid, with a high test-retest reliability of > 0.74 (Zbogar et al. 2016). Depending on the severity/completeness of the injury, participants were grouped according to mobility status and were classified as either wheelchair users or ambulatory individuals. Accelerometer cut-points were then used to analyse different PA intensity categories based on the vector magnitude of the output from the accelerometer, where three intensity categories were generated for wheelchair users (sedentary behaviour [SED], light intensity PA [LIPA] and moderate-to-vigorous PA [MVPA]) and two intensity categories for ambulatory individuals (SED/LIPA and MVPA). This was based on cut-points derived from the literature (Bammann et al. 2021; Holmlund et al. 2019), where currently, there are no cut-points available for ambulant adults with TSCIs (Table 1). Thus, the cut-points used for ambulatory individuals were derived from a study of elderly abled-bodied individuals aged 59–73 years (Bammann et al. 2021). Ambulant adults with SCI may be experiencing mild impairments related to gait and walking speed, and these changes could correlate to age-related changes experienced by elderly, able-bodied individuals. This provides the rationale for the use of these cut-points.

**TABLE 1:** Physical activity levels: Cut-points.

Subgroup	Sedentary behaviour (VMC/min)	Light-intensity PA (VMC/min)	Moderate PA (VMC/min)	Vigorous PA (VMC/min)
**Wheelchair users**				
Tetraplegia	< 2000	3462–4886	4887–9278	9279
Paraplegia	< 2000	6997–9514	9515–13 238	13 239
**Ambulatory individuals**				
Male	-	< 8828	8829	12 482
Female	-	< 9364	9365	20 962

PA, physical activity; VMC, vector magnitude counts.

### Statistical analysis

Data were captured, summarised and visualised into an Excel spreadsheet for all outcome measures. Data were then coded and transferred to SPSS v.28 software for analysis. Demographic, injury characteristics and outcome measures indices were displayed using descriptive statistics and presented using frequency tables, means and medians where necessary. The accelerometer data were downloaded using the ActiLife 6 software and subsequently analysed. When downloading the data, an AGD file was created, and data were analysed in 60 s epochs. These data were analysed according to each daily segment and presented as time spent in each intensity category. The cut-points retrieved from previous literature were used to classify intensity categories (Bammann et al. 2021; Holmlund et al. 2019). Data were then presented as time spent engaged in SED/LIPA and MVPA for ambulatory individuals, while wheelchair users’ data were presented as SED, LIPA and MVPA. Following the description of PA according to intensity levels, independent sample t-tests were conducted to determine whether any known group differences exist in PA intensity by mobility status (wheelchair users and ambulatory individuals, respectively), gender (male vs. female), age (younger vs. older) and years lived with SCI (shorter vs. longer).

### Ethical considerations

Ethical clearance for our study was obtained from the University of the Western Cape’s Biomedical Research Ethics Committee (reference no.: BM19/7/2) and the GSH Research Board. All participants provided written informed consent in a language of their choice before the commencement of our study. A unique code replaced all participants’ identification, and all data stored on a password-protected device to optimise and ensure confidentiality.

## Results

A total of 76 participants with TSCI were included in our study, and their socio-demographic and injury characteristics, as well as functioning indices, are presented in Table 2. Most of the participants were male (*n* = 67, 88.2%), with a mean age of 36 years for the entire cohort. The most common cause of injury was because of assault (*n* = 49, 64.5%). At the time of injury, 65.8% (*n* = 50) reported being employed with a corresponding 15.8% (*n* = 12) being employed after sustaining their TSCI. The secondary complication’s mean total score was 19.18 ± 7.30, while the SCIM-SR mean score was 57.22 ± 23.41. Participants’ socio-demographic and injury features are displayed in Table 2.

**TABLE 2:** Participants’ characteristics of study population (*N* = 76).

Variables and outcomes	*n*	%	Mean	s.d.	Median	IQR
**Sociodemographic characteristics**						
Age (years)	-	-	36	10.93	-	-
Male gender	67	88.2	-	-	-	-
Living: family	58	76.4	-	-	-	-
Wheelchair user	62	81.6	-	-	-	-
Employed (time of injury)	50	65.8	-	-	-	-
Unemployed (current)	64	84.2	-	-	-	-
**Injury characteristics**						
Paraplegia	40	52.6	-	-	-	-
AIS A	38	50.0	-	-	-	-
Assault	49	64.5	-	-	-	-
Time since injury (3–6 years)	59	77.6	-	-	-	-
**Personal CVD risk factors**						
Physically inactive	51	67.1	-	-	-	-
Increased body mass index	22	28.9	-	-	-	-
Hypertension	6	7.9	-	-	-	-
Diabetes mellitus, Cholesterol	2	2.6	-	-	-	-
**Number of CVD risk factors**						
None	23	30.3	-	-	-	-
1 risk factor	28	36.9	-	-	-	-
2 risk factors	22	28.9	-	-	-	-
3 risk factors	3	3.9	-	-	-	-
**Family history of CVD risk factors**						
Heart disease	17	22.4	-	-	-	-
Vascular disease	13	17.1	-	-	-	-
Cerebrovascular accident	11	14.5	-	-	-	-
**Body functions and activity**						
Secondary complications (range 0–48)	-	-	19.18	7.30	-	-
SCIM-SR: Self-care subscale (range 0–20)	-	-	15.03	6.95	-	-
SCIM-SR: Respiratory and sphincter management subscale (range 10–40)	-	-	-	-	25.50	18.00
SCIM-SR: Mobility subscale (range 0–40)	-	-	16.46	9.90	-	-
SCIM-SR: Total scores (range 10–100)	-	-	57.22	23.41	-	-

s.d., standard deviation; CVD, cardiovascular disease; IQR, interquartile range; SCIM-SR, Spinal Cord Injury Measure – self-report.

### Objectively-assessed physical activity

Seven participants (9%) had invalid accelerometer data (had not adhered to the stipulated wear time protocol) and were therefore excluded from further analysis. The average total wear time for wheelchair users (*n* = 56) was 981.19 min/day and 991.27 min/day for ambulatory individuals (*n* = 13). Participants, both wheelchair users and ambulatory individuals, spent the majority of their time engaged in SED and LIPA, followed by MVPA. Ambulatory individuals spent 98.1% of their time engaged in SED/LIPA. In comparison, wheelchair users spent 77.6% in SED and 20.7% for LIPA while less time was spent in MVPA for both wheelchair users and ambulatory individuals (Table 3).

**TABLE 3:** Participants’ objectively assessed physical activity levels according to intensity levels (*N* = 69).

Variables	Wheelchair users (*n* = 56)	Ambulatory individuals (*n* = 13)
Wear time (min/day)	981.19	991.27
**Volume of PA**		
Vector magnitude	1209424.73	679286.75
**SED/LIPA**		
Minutes per day (mean)	-	972.47
Percentage of wear time	-	98.10%
**Sedentary**		
Minutes per day (mean)	761.12	-
Percentage of wear time	77.60%	-
**LIPA**		
Minutes per day (mean)	203.11	-
Percentage of wear time	20.70%	-
**MVPA**		
Minutes per day (mean)	16.96	18.80
Percentage of wear time	1.70%	1.90%

PA, physical activity; SED, sedentary behaviour; LIPA, light intensity physical activity; MVPA, moderate-to-vigorous physical activity.

### Demographic and injury duration factors associated with physical activity intensity levels

The exploratory analytical analyses revealed a significant (*p* = 0.005) difference in time spent in MVPA concerning years living with TSCI for ambulatory individuals, where those affected with the injury for a shorter period were more active in MVPA (Table 4). Furthermore, among wheelchair users, those in the younger category (18–39 vs. 40–67) spend significantly (*p* < 0.001) less time in MVPA than their older counterparts (Table 5). No known group differences were found for ambulatory individuals (Table 4) and wheelchair users (Table 5).

**TABLE 4:** Comparison of physical activity intensities across the subgroup of the spinal cord injury population: Ambulatory individuals (*N* = 69).

Measures	*n*	SED/LIPA (mean)	*T*	*p*	MVPA (mean)	*T*	*p*
**Gender**	-	-	−1.300	0.984	-	0.849	0.230
Male	11	952.21	-	-	22.04	-	-
Female	2	1083.93	-	-	1.00	-	-
**Years with SCI**	-	-	−0.737	0.260	-	1.443*	0.005*
1–3 years	6	941.98	-	-	32.00	-	-
4–6 years	7	998.61	-	-	7.49	-	-
**Age (years)**	-	-	−1.761	0.529		1.137	0.118
18–39	10	939.01	-	-	24.24	-	-
40–67	3	1084.00	-	-	0.67	-	-

SED, sedentary behaviour; LIPA, light intensity physical activity; MVPA, moderate-to-vigorous physical activity; SCI, spinal cord injury.

* , significant at *p* < 0.05.

**TABLE 5:** Comparison of physical activity intensities across subgroup of the spinal cord injury population: Wheelchair users (*N* =69).

Measures	*n*	Sedentary			LIPA			MVPA		
		Mean	*T*	*p*	Mean	*T*	*p*	Mean	*T*	*P*
**Gender**	-	-	0.741	0.783	-	−0.397	0.068	-	−0.818	0.291
Male	49	766.48	-	-	200.25	-	-	15.67	-	-
Female	7	723.62	-	-	223.16	-	-	26.01	-	-
**Years with SCI**	-	-	−0.333	0.476		0.570	0.062		0.055	0.785
1–3	23	753.46	-	-	216.10	-	-	17.24	-	-
4–6	33	766.46	-	-	194.06	-	-	16.77	-	-
**Age (years)**	-	-	0.324	0.782	-	−0.163	0.830	-	−2.909*	< 0.001*
18–39	43	757.70	-	-	201.40	-	-	10.71	-	-
40–67	13	772.43	-	-	208.78	-	-	37.64	-	-

LIPA, light intensity physical activity; MVPA, moderate-to-vigorous physical activity; SCI, spinal cord injury.

* , significant at *p* < 0.05.

## Discussion

Our study described the time spent in different PA intensities among adults with TSCIs living in the community. It investigated whether associations exist between demographic and injury duration characteristics and PA intensity. Our study forms the foundation for understanding how PA should be supported and tailored in a South African context.

Primarily, our study found that adults with TSCIs spent an alarming amount of time in SED and LIPA for both wheelchair users and ambulatory individuals. According to previous literature, SED is characterised as any waking activities performed while in a seated or reclined position that results in an energy expenditure of ≤ 1.5 metabolic equivalents (METs), while LIPA includes activities such as slow inside walking, household tasks requiring light effort (i.e. making the bed, sweeping the floors, dusting, eating and preparing food, hairstyling) or wheelchair propelling on tiles; that results in an energy expenditure < 3 METs (Ainsworth et al. 2000; Bates et al. 2022; Collins et al. 2010; Gibbs et al. 2015; Veerubhotla et al. 2020). The increase in SED and LIPA after an SCI corroborates with previous studies, which also found a decline in PA levels, especially for wheelchair users (Warms et al. 2008). This was seen in a study by Warms et al. (2008) that reported more than 9 h spent in bed and/or sleeping and 12 h spent in LIPA daily. Adults with SCIs, as well as healthcare providers not educated or trained in SCI management, may not understand the importance or benefits of PA and may believe that resting is necessary to conserve energy and decrease pain (Warms et al. 2008). It is, however, also evident that different activities and tasks in daily life produce different energy expenditures, which is still largely unknown to adults with SCIs and healthcare professionals alike (Ainsworth et al. 2000; Warms et al. 2008). A health promotion intervention is required to educate adults with SCIs on the type of activities that could be performed daily to induce different intensity and exertion levels, which, in turn, would benefit for them from a PA and health-enhancing perspective. These everyday and meaningful activities should be introduced and supported during inpatient rehabilitation as a means of starting with PA and exercise prescription as a crucial therapeutic target in all phases of care.

For adults with SCIs, maintaining an active lifestyle has shown to be challenging, especially once they have reintegrated back into their communities following an intensive inpatient rehabilitation (Mat Rosly et al. 2018). Efforts to increase PA participation within the SCI community are often impeded by a lack of motivation, physical impairments, institutional and policy barriers as well as a lack of accessibility (Mat Rosly et al. 2018; Paulus-Mokgachane, Visagie & Mji 2019). Generally, ambulant adults with SCI seem to be better at engaging in PA once they are home, as they can stand and walk and partake in ADLs such as house chores, self-care and move around their communities with less limitations than someone using a wheelchair (Warms et al. 2008). These findings were evident in a longitudinal study performed in the Netherlands that followed a group of participants, from inpatient rehabilitation and into the community 1-year post rehabilitation and discovered that PA levels increased from 88.7 ± 36.1 min/day to 116 ± 59.2 min/day and SED decreased from 732.4 ± 81.9 min/day to 665 ± 121.3 min/day. These findings still, however, revealed an alarming time spent in SED and LIPA (Postma et al. 2020). The increase in PA levels was related to an increase in walking mobility within the community post-discharge (Postma et al. 2020). Our study revealed similar findings where ambulatory individuals engaged in more PA, but much time spent in MVPA (18.80 min/day). Various factors could influence the decrease in activity levels seen in ambulant participants in our study, which should be taken into consideration. Factors, such as safety and gang-related violence in communities (high incidence of assault with this cohort) impacting vulnerability, infrastructure and environmental challenges, walking speed, availability of an appropriate or use of an assistive device or attitudes of community members, are possibilities for poor PA levels and increased SED within the local context (Mat Rosly et al. 2018; Paulus-Mokgachane et al. 2019; Warms et al. 2008). Thus, there is a need to understand both intrinsic and extrinsic factors related to PA levels in general, including frequency, duration and intensity.

Our study further explored whether relationships exists between demographic variables such as gender, age and time since injury, as well as time spent in different PA intensity categories. A significant finding was seen with older wheelchair users (age: 40–67 years) demonstrating more engagement in MVPA than the younger age group. The results of our study may provide a hopeful possibility that participants using wheelchairs to aid their mobility are finding ways to be more active within their home and community environments and including PA into their daily routines. However, older participants using wheelchairs depending on the severity of the injury, require more effort and may exert more energy to complete ADLs, giving the impression of higher engagement in MVPA. These findings were similarly seen in a study done by Watson et al. (2022) where older males engaged in more MVPA compared to their counterparts. South Africa’s unique SCI profile reveals that we have a predominantly younger male cohort, with majority aged between 18 and 29 years (Joseph 2017; Madasa et al. 2020; Njoki, Frantz & Mpofu 2007), and this could provide a possible reason for our cohort demonstrating lower levels of MVPA and more time spent SED. This may be suggestive of a potential barrier to PA levels within the local setting. Although our study did not look at factors associated with PA levels, previous literature has identified themes such as intrapersonal, interpersonal, policy and community levels as common perceived barriers to PA (Mat Rosly et al. 2018; Vermaak et al. 2022).

In addition, varying findings are seen in previous literature that explores time since injury and PA engagement. As with our study, it was found that adults living with more recent injuries engaged in more PA compared to chronic injuries, while other studies found increased involvement in PA for participants living with their injuries for a longer time (Mat Rosly et al. 2018; Postma et al. 2020). Even though this factor revealed a significant finding for MVPA, the time spent in MVPA was minimal and did not meet the global recommendations for PA from the WHO, which is a big concern as it is an important modifiable risk factor for CVD and potentially other health conditions. While little is known about the availability of rehabilitation services for SCIs within the communities, intensive rehabilitation is offered by a specialised unit in the Western Cape, which could account for the increased participation observed in those with more recent injuries. The PA findings in this cohort corroborate with previous studies that report alarming time spent in SED and LIPA and poor engagement in MVPA (Eitivipart et al. 2021; Mat Rosly et al. 2018). The common findings seen across literature on measuring PA levels are that adults with SCI are not meeting the recommended guidelines for PA to achieve beneficial health-enhancing outcomes. We can also conclude from these findings that this cohort does not meet recommended levels of PA.

Engaging in MVPA is known to reduce the risk of CVD, prevent or decrease the development of secondary complications and improve overall physical fitness and quality of life in adults with SCI. Therefore, promoting an active lifestyle is imperative for this population, and understanding modifiable influencing conditions would be the first step to develop novel interventions and strategies to promote PA in the future. Future studies should explore how PA and exercise behaviour evolve over the course of living with a TSCI, as well as understand how PA can be supported and made accessible at all levels of healthcare. Furthermore, healthcare professionals should be sensitised and equipped to provide information and advice on becoming and/or maintaining one’s PA levels and exercise and fitness levels once confronted with a TSCI.

### Strengths and limitations

To our knowledge, this is the first study to objectively measure PA in adults with SCIs in South Africa. Participants were adherent to our study protocol, and most of them wore the device for the prescribed time; however, a 9% dropout rate was found. Post hoc analysis revealed that no differences in demographic and injury characteristics were found between those who dropped out versus those completing our study, which enhances the generalisability of the findings to the included sample. It is important to observe as well is the small sample size and use of convenient sampling, which hinder generalisability of the findings to the broader SCI population. As previously said a large proportion of participants were unreachable, and this has resulted in an underpowered sample size; however, the research findings are still valuable as this is the first descriptive study performed on PA for this cohort in the local setting.

In addition, because of device constraints, each participant was fitted with one device. There is a possibility that upper limb ADLs may not have been detected with ambulatory individuals who had the device anchored around the ankle, therefore underestimating PA levels of the upper body/limbs. For future studies, it is recommended to have participants fitted with a wrist and ankle monitor as well as including a subjective measurement tool, which could add valuable information about PA levels.

There are no SCI-specific cut-points for ankle-worn activity monitors available, and therefore, the most appropriate cut-points were retrieved from previous literature but were specific to older aged able-bodied individuals. These cut-points for elderly persons were deemed appropriate as ambulant adults with TSCI could be experiencing similar impairments related to gait, speed, etc. This is, however, not the golden standard but still provides insights into intensities with a comparable reference group. In addition, a need exists to determine the energy expenditure of ambulatory individuals with SCI to derive calibrated cut-points specific to the population. Lastly, our study was underpowered in terms of females and ambulatory individuals. A better representation of these adults with TSCI is needed to fully assess their impact on PA levels in the local setting. Having more equal representation of minority sub-groups of SCI is essential for ensuring that any solution or intervention is applicable to all individuals.

### Recommendations

- Future research should focus on identifying and understanding the determinants of PA levels for the TSCI population, with specific reference to personal, environmental and systemic barriers.
- Longitudinal studies should be conducted to ascertain the direction of the relationship between exposures and outcomes, such as PA, and could be conducted at different time intervals to determine how these characteristics and outcomes evolve over time.
- Interventional studies with a primary focus on testing tailored strategies to increase engagement of MVPA, such as community-based programmes, digital health solutions and individualised exercise prescriptions should be conducted, but initially, patient preferences for exercise and PA should be determined.
- Future research exploring the impact of meeting PA guidelines with specific health outcomes, such as cardiovascular health, mental well-being and quality of life, would provide the necessary evidence to support advocacy and policymaking for enhanced PA promotion in the SCI population.

### Implications of findings

The PA is compromised after an SCI, and this could lead to increased weight gain and an increased risk of CVD, which has become a leading cause of death in developed countries. Having comorbidities coupled with poor fitness limits function, community and social participation. Little is known about PA levels among the SCI population. Now, the results of our current research have identified that community living adults with SCIs are spending an alarming time in SED followed by LIPA and not meeting the minimum requirements needed for health-enhancing benefits. This research provides valuable information needed to show the importance of PA, which could be used to influence improvements in healthcare services aligned with PA promotion. Furthermore, information gained with this research could support the development of future PA promotion interventions.

## Conclusion

Our study highlights that adults with TSCIs spent most of their time in SED and LIPA with minimal engagement in MVPA. The findings further indicated that time spent in MVPA was significantly more among adults with more recent injuries as well as older participants, demonstrating variations in subgroups, which could warrant further investigation. It is evident that adults with TSCIs are not meeting recommended PA guidelines for beneficial health outcomes. There is thus a need to investigate the determinants of PA levels to arrive at modifiable intervention targets.
